# Pathway results from the chicken data set using GOTM, Pathway Studio and Ingenuity softwares

**DOI:** 10.1186/1753-6561-3-S4-S11

**Published:** 2009-07-16

**Authors:** Agnès Bonnet, Sandrine Lagarrigue, Laurence Liaubet, Christèle Robert-Granié, Magali SanCristobal, Gwenola Tosser-Klopp

**Affiliations:** 1 INRA, UMR444, Laboratoire de Génétique Cellulaire, F-31326 Castanet-Tolosan, France; 2 INRA, UMR 598, Génétique Animale, F-35000 Rennes, France; 3 Agrocampus Ouest, UMR 598 Génétique Animale, F-35000 Rennes, France; 4 INRA, UR631, Station d'Amélioration Génétique des Animaux, F-31326 Castanet-Tolosan, France

## Abstract

**Background:**

As presented in the introduction paper, three sets of differentially regulated genes were found after the analysis of the chicken infection data set from EADGENE. Different methods were used to interpret these results.

**Results:**

GOTM, Pathway Studio and Ingenuity softwares were used to investigate the three lists of genes. The three softwares allowed the analysis of the data and highlighted different networks. However, only one set of genes, showing a differential expression between primary and secondary response gave significant biological interpretation.

**Conclusion:**

Combining these databases that were developed independently on different annotation sources supplies a useful tool for a global biological interpretation of microarray data, even if they may contain some imperfections (e.g. gene not or not well annotated).

## Background

Microarray data usually provide lists of genes, and often of hundreds of genes. The challenge is next to give sense to these lists and help to interpret the results. We used the three lists of differentially expressed genes presented in the introductive paper [1] as examples of the way to do such analyses. They address three different biological questions: primary or secondary response effect (MM8-PM8, 1736 genes), species effect (MM8-MA8, 85 genes) and time effect (MM8-MM24, 800 genes).

The biological interpretation of gene lists was performed by three approaches: study of GO enrichment terms with GOTM (Gene Ontology Tree Machine) and two pathway software with a demonstration version of Pathway Studio (Ariadne Genomics) and Ingenuity Pathway Analysis (IPA, Ingenuity Systems Inc., Redwood City, CA). These approaches used various gene annotation databases: 1-Gene Ontology (GO) describes the most important ontologies in molecular biology, 2-Pathway Studio database is curated electronically using automated text-mining engines that are also available to investigators and, 3-Ingenuity's knowledge base is the first large database developed on all types of gene-gene and metabolite-gene interrelationships extracted manually from publications.

The purpose of our study is to evaluate three different *in silico *approaches in order to give sense to gene lists from microarray data.

## Methods

### GOTM

Gene ontology (GO) [2] constitutes a controlled vocabulary of about 20,000 terms organized in three independent hierarchies for cellular components, molecular functions, and biological processes [3]. The GO analyses of clusters were performed using the Gene Ontology Tree Machine (GOTM) software [4]. The GOTM web-based tool supplies statistical analysis to identify enriched Gene Ontology categories for the input gene sets and generates a GOTree, a tree-like structure to navigate the Gene Ontology Directed Acyclic Graph to help users visualize the GO-term relations. Hypergeometric test was used to select enriched GO terms for each cluster compared to the GO terms of the annotated genes present on the microarray (10451 genes, in this study). A GO category was considered as enriched for a level of p-value < 0.01.

### Pathway Studio

We run the "Subnetwork Enrichment Analysis" (SNEA) option of the software, with default parameters, which builds small networks consisting of a single "regulator" gene and its targets [5]. The significance of the target expression levels in every built network is evaluated next. The algorithm finds the individual "regulators" which most likely affect differentially expressed targets. Thus, it is expected to provide the most plausible explanation for the observed expression changes. We then built the union of pathways from the selected networks.

### Ingenuity Pathways Analysis

This system, a web-based interface [6], provides computational algorithms to identify and dynamically generate significant biological networks and pathways that are particularly enriched with our genes of interest called "focus genes". It also ranks networks by a score that takes into account the number of focus genes and the size of the networks, indicating the likelihood of the focus genes in a network being found together by chance. The higher the score (score = -log(p-value)), the lower is the probability of finding the observed Network Eligible Molecules in a given network by chance. IPA also gives information on biological functions and canonical pathways. The data set contains the three differentially expressed gene lists and the microarray gene list. It includes a column with mixed identifiers (HUGO symbols, REFSEQ...) and a column with gene fold changes. This data set was uploaded as a tab-delimited text file. We compared the 3 gene lists to the list of genes of the microarray and underlined the specific enrichment of regulated genes.

## Results

### GOTM

Among the 11538 oligonucleotides of the microarray with a proposed HUGO name, 10451 HUGO gene names were validated according to HGNC [7]. The HUGO gene name is a unique human ortholog and an abbreviation name provided by the annotation EADGENE Network of Excellence, funded by the EC and available on [8].

For the 3 condition contrasts MM8-MA8, MM8-MM24 and MM8-PM8, the gene numbers with validated HUGO among the differentially expressed genes were 38 among 85, 354 among 800 and 931 among 1736 respectively. Because GOTM does not use more than 500 genes at once, we had to split the MM8-PM8 list into 2 sets: up and down regulated genes (Table 1). Only the biological process GO category was reported in the present study (p-value < 0.01). As indicated in Table 2, six biological processes were enriched in MM8-MM24 and MM8-MA8 sets. Concerning the MM8-PM8 experimental condition, six and seven biological processes were enriched for the down-regulated and up-regulated gene sets respectively.

**Table 1:** Number of validated identifiers with the three softwares

	MM8-MA8	MM8-MM24	MM8-PM8
Number of validated identifiers	85	800	1736
GOTM	38	354	931
Pathway Studio	42	382	1037
Ingenuity	43	386	1062

**Table 2:** Enriched biological process GO terms (P < 0.01) obtained by the Gene Ontology Tree Machine (GOTM).

Enriched biological process GO terms	P < 0.01	Time effect	Species effect	Injection effect
organelle organization and biogenesis:	P = 0.005			
ARPC3 KATNB1 DCTN6 KIF3A JMJD2A HDAC4 MTSS1				
CBX3 ADRB2 TIMM8A GOLGB1 RHOA RHOB NEDD9 SPTBN5 PEX1 H2AFY2 FMN2 RHOJ		MM8-MM24	MM8-MA8	
EP400 NSD1 SOX2 BRCA2 YWHAH DDX54 NOC4L ATG9A SUV39H2 SMC3 CDC42BPB				
gluconeogenesis:	P = 0.008			
ATF4 ACN9 TPI1				

glutamine biosynthesis:	P = 0.005			
GLUL CORO2A				
nervous system development:	P = 0.005	MM8-MM24		
SPON2 NCKAP1 CHRNA4 CNTF DAB1 TIMM8A EFNB1 NEUROG1 PAX5 RPS6KA3				
YWHAH SEMA6D ST8SIA2 PARD6B LGI1 NOG CD9 ECE2 HDAC4 MTSS1				

organelle organization and biogenesis:	P = 0.009			
NSD1 L3MBTL DST NEDD9 SPTBN5 PFN2			MM8-MA8	
pyruvate metabolism:	P = 0.001			
ATF4 TPI1				

G-protein signaling\, coupled to IP3 second messenger phospholipase C activating:	P = 0.008			
NMUR1 DGKG P2RY4 AVPR1A DGKZ SPHK1				
UDP-N-acetylglucosamine metabolism:	P = 0.006			
UAP1 GNE				
negative regulation of DNA replication:	P = 0.0009			MM8-PM8
ENPP7 S100A11 CDT1				
endocytosis:	P = 0.0047			genes down-regulated
ADRB2 AP1B1 DOCK1 PACSIN3 PACSIN1 LRP6 NECAP2 ELMO3 SNX4				
tissue development:	P = 0.004			
APOA5 EDA ELF3 LAMB3 LAMC2 BMP4 SOX9 TFAP2A TGFB2 TUFT1				
ectoderm development	P = 0.003			
EDA ELF3 LAMB3 LAMC2 TFAP2A TGFB2				

immune response-regulating signal transduction:	P = 0.003			
FYN SPG21 PTPRC				
caspase activation:	P = 0.004			
DIABLO STAT1 CASP9 CASP8AP2				
Golgi vesicle transport:	P = 0.006			
SEC23A EXOC5 TMED2 COPE COPB1 SEC22A SAR1A MCFD2 COPB2 GOSR1				MM8-PM8
protein targeting to mitochondrion:	P = 0.008			genes up-regulated
TIMM44 TOMM34 TSPO GRPEL1				
chromatin assembly or disassembly:	P = 0.002			
CBX3 HMGB2 HP1BP3 HDAC8 SMARCE1 CHAF1B SMARCA5 TLK1				
modification-dependent protein catabolism:	P = 0.008			
FBXO21 RNF11 PSMA2 PSMA5 PSMB1 PSMB3 PSMC5 UBE2E1 UBE3A USP7 PSMF1 USP3				
protein transport:	P = 0.002			
34 genes				

For each set of data, the enriched biological processes found with GOTM analysis are listed, with their probability and representative genes.

### Pathway Studio

For the 3 condition contrasts MM8-MA8, MM8-MM24 and MM8-PM8, the gene numbers mapped with the software were 42 among 85, 382 among 800 and 1037 among 1736 respectively (Table 1).

**Figure 1:**
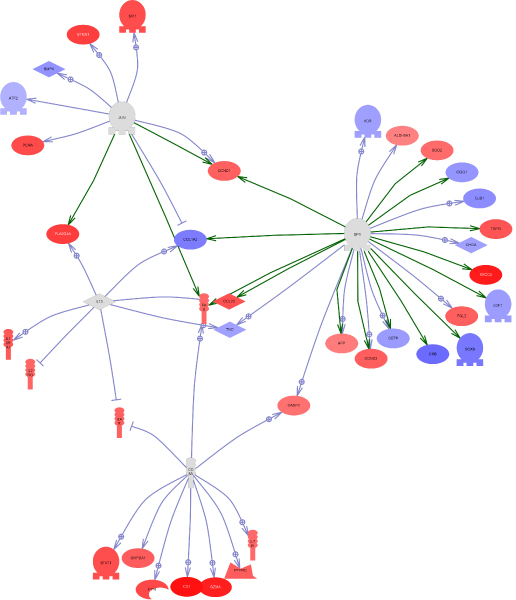
**Combined pathway from the Sub Network Enrichment Analysis of Pathway Studio (primary or secondary response effect)**. Molecules are identified with their HUGO symbol. Red colour shows up regulated genes in the PM8 (primary response) condition versus MM8 (secondary response). Blue colour shows down regulated genes in the PM8 (primary response) condition versus MM8 (secondary response).

The SNEA analysis gave significant results only for the MM8-PM8 gene list. Expression targets of JUN, CD8A, IL13 and SP1 were found as significantly over-represented (p-value <0.05). The combined pathway is represented in Figure 1. The software allowed to visualise the regulation of the target genes in this combined pathway, using the two other lists of genes (MM8-MA8) and (MM8-MM24) and showed 1 to 4 regulated targets (data not shown).

### Ingenuity

Among the 13639 annotated genes from the microarray using the different identifiers, 12395 are recognized by the software and 7219 used to generate networks. For the 3 condition contrasts MM8-MA8, MM8-MM24 and MM8-PM8, the gene numbers mapped with Pathway Studio were 43 among 85, 396 among 800 and 1062 among 1736 respectively (Table 1).

First, we searched to identify the networks, functions or canonical pathways from the microarray gene list. One hundred networks are built from the whole microarray with a maximum score of 18. This score obtained for the microarray gene list is considered as a threshold and is used as the minimum score accepted to define a network for the next 3 gene list as significantly enriched.

Then, Ingenuity gives 22 significant enriched networks from the 3 gene lists with a score between 19 and 55 (Additional datafile 1). The enriched functions and canonical pathways were presented in Additional datafile 2.

**Figure 2:**
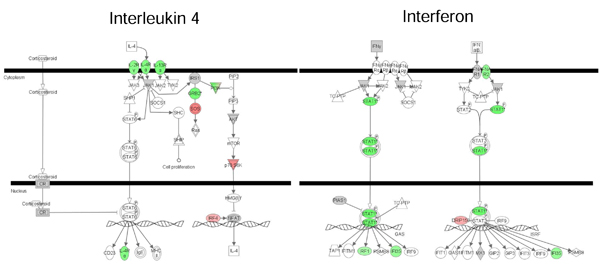
**Canonical pathway underlines genes involved in inflammatory cytokines signalisation from the primary/secondary response comparison (PM8-MM8)**. We compared the secondary infection to the single ones. Green colour shows down regulated genes in the MM8 (secondary response) condition versus PM8 (primary response). Red colour shows up regulated genes in the MM8 (secondary response) condition versus PM8 (primary response).

Ingenuity underlines:

a) In the PM8-MM8 comparison, 14 networks are involved in significant biological functions compared to the microarray background. Biological functions as immune and inflammatory response, metabolism and proliferation are enriched. Particularly, the merged immunity network shows the down expression of a majority of genes during the secondary response. In accordance with previous results [9], canonical pathway shows specifically the down expression of genes involved in inflammatory cytokines signalisation as interleukin 4 and interferon signalisation during the secondary infection (Figure 2).

b) In the MM8-MM24 comparison, 3 networks (out of 7) and the canonical pathways results show mostly a down expression of the genes involved in the actin polymerisation.

c) In the MM8-MA8 comparison, only 1 significant network (score 22) was identified with down expressed genes involved in inflammatory diseases in *Eimeria acervulina *species compared to *Eimeria maxima*.

## Conclusion

In this study, we used 3 lists of genes with 3 softwares. It is noticeable that slight differences (+/-10%) exist between the 3 softwares concerning the number of mapped genes. We identified two limits, using these programs: first, GOTM did not allow us to study all the genes at once; second, whatever program we used, the number of genes with an accepted name never reached 100% of the annotated list. The capability of the software to identify the gene names is a limiting step of the analysis.

Only primary vs. secondary response gene list (MM8-PM8) gave significant biological interpretation with the 3 softwares: immune function and inflammatory response were much higher in primary response (immune-system related processes).

Because these databases were developed on different annotation sources, the results can be considered as complementary. Combining these databases supplies a useful tool for a global biological interpretation of microarray data, even if they may contain some imperfections (gene not or not well annotated).

In another way, GOTM offers more accurate information for a list of genes but did not suggest any links between these genes. GOTM is more appropriate for description of the data with a statistical validation.

Pathway Studio, in the way it was used in this work, presents two main characteristics that differentiate it from the other softwares. First, it tries to find links only between the genes in the proposed list and not in a more global context like Ingenuity. Secondly, it offers the possibility to identify major regulators for differentially regulated genes and this could be interesting in order to develop further studies. The proposed networks look like stars with the possible regulator in the node.

Ingenuity suggests more possible links between the differentially regulated genes and with some other genes in highly relevant networks. The networks are more complex than with Pathway Studio with more nodes, more genes without information about their expression. Ingenuity explores list of genes by very different ways, for example as very high confident information with canonical pathways and more exploratory ways as gene network.

These softwares give tools to functionally annotate the gene lists and help the biologists to give sense to huge data. Finally, these softwares offer the possibility to identify more interesting genes among a list in order to undertake further experiments.

## Competing interests

The authors declare that they have no competing interests.

## Authors' contributions

AB carried out the Ingenuity study, SL carried out the GOTM study. GTK carried out the Pathway Studio study and drafted the manuscript. All authors participated to a working group, the redaction work, read and approved the final manuscript.

## Supplementary Material

Additional file 1 **IPA networks**. For each of the 3 lists the networks are selected if their score is higher than 21 (the higher network score value generated by all the microarray genes (score >18). The table contains columns with the network number, the name of the comparison list, the names of the genes involved in the network, the score value and the top functions. Click here for file

Additional file 2 Data were filtered with 2 criterions: IPA threshold p.value (<0.05) and the corresponding microarray p.value. a – Biofunctions: we did not find significant functions for the MM8-MA8 gene list. b – Canonical Pathway. For each list, the pathways are ranked by the score (score = -log(p.value)) using the same criterions. The table includes also the ratio (number of focus molecules in a given pathway divided by the total number of the molecules that makes up that pathway). Click here for file
