# Supplementary material for: Pathway results from the chicken data set using GOTM, Pathway Studio and Ingenuity softwares
Source: BMC Proc. 2009 Jul 16;3(Suppl 4):S11. doi: 10.1186/1753-6561-3-S4-S11 (PMC2712741; doi:10.1186/1753-6561-3-S4-S11)
Supplement: Additional file 1 — IPA networks. For each of the 3 lists the networks are selected if their score is higher than 21 (the higher network score value generated by all the microarray genes (score >18). The table contains columns with the network number, the name of the comparison list, the names of the genes involved in the network, the score value and the top functions. [file 1753-6561-3-S4-S11-S1.doc]

Supplemental Data 1 : IPA networks

For each of the 3 lists the networks are selected if their score is higher than 21 (the higher network score value generated by all the microarray genes (score >18).

The table contains columns with the network number, the name of the comparison list, the names of the genes involved in the network, the score value and the top functions.

| ID | Analysis | Molecules in Network | Score | Focus Molecules | Top Functions |
| --- | --- | --- | --- | --- | --- |
| 1 | MM8-MM24 | APOA5, ATF4, BRCA2, C19ORF2, CBX3, CCDC71, COL18A1, COL1A2, CREB5, CUX1, Cyclin A, DLL4, FGB, FMR1, Histone h3, IRF4, MMP2, NEUROG1, NFKB1, NID1, NID2, NSDHL, PPP1R12A, PSMA2, Ras homolog, RFX2, RHOA, RHOB, RHOJ, RNA polymerase II, RPS6KA3 (includes EG:6197), SREBF2, STAR, TFAP2A, USF1 | 55 | 31 | Cardiovascular System Development and Function, Cell Morphology, Cellular Development |
| 2 | MM8-MM24 | ABL2, ADRB2, Akt, AKTIP, Beta Arrestin, CACNA1C, CALD1, Calmodulin, CFLAR, DUSP1, ERK, F Actin, GDI2, HIP1R, HTR1A, MAP2K3, MAP2K5, MAP3K4, MAPK10, Mek, MTSS1, MVP, P38 MAPK, PAK2, PKN2, Rac, RNF11, SIVA1, SORBS2, STAM2, TEK, TIMM8A, TNIP1, TRPV4, VPS37C | 44 | 27 | Cell Morphology, Cell-To-Cell Signaling and Interaction, Cellular Assembly and Organization |
| 3 | MM8-MM24 | A2M, ADAMTS7, CD9, COIL, COX15, CREB3L3, CYB5R2, Cytochrome c oxidase, E2F1, EIF1, HDAC1 (includes EG:3065), IFRD1, IGBP1, JUN, LPCAT1, LRRTM4, MAFB, MID1, MID2, Mmp, MMP15, MMP24, NCAM1, NF-E4, NOVA1, PALM, PAX5, PAX6, RB1, REST, SAP30, SLC16A10, ST8SIA2, TFCP2, USP48 | 27 | 19 | Gene Expression, Cell Cycle, Nervous System Development and Function |
| 4 | MM8-MM24 | Actin, ADRB1, ADRB2, ANKRA2, ANKRD11, CAMK4, Cbp/p300, CHRNA4, CHRNG, CTLA4, EFNB1, EP400, Ephb, FLT3, GRIN3A, HDAC4, IRF, IRF5, IRS, MDM2 (includes EG:4193), NEK1, NFAT complex, NFkB, OGG1, PARD6B, PARD6G, PI3K, PIK3R1, PP2A, PPP2R4, S100A11 (includes EG:6282), SMPD1, TEK, YWHAH, YWHAQ (includes EG:22630) | 25 | 18 | Cancer, Ophthalmic Disease, Renal and Urological Disease |
| 5 | MM8-MM24 | ARHGDIA, ATP5I, ATP6V1F, AVPR1A, CDC42, CDC42BPB, CREB1, DLG4, DUSP12, ERBB2, EXOC1, EXOC3, EXOC4, EXOC5, FNBP1L, GRK5, HNF4A, Hsp90, IHPK2, KLHL17, LRIG1, LRRC1, MAPK1, PDZK1, PDZK1IP1, PLXNA1, PTK7, RIN2, SCNN1G, SDK2, SEMA3C, SEMA6D, SIPA1L1, ST3GAL4, SYNGAP1 | 25 | 18 | Cell Morphology, Cellular Assembly and Organization, Nervous System Development and Function |
| 6 | MM8-MM24 | ABHD6, ACN9, ARL1, C20ORF116, C4ORF34, CDK5RAP3 (includes EG:80279), ECE2, GHITM, GSTK1, GTF2I, HDAC3, HNF4A, HSPA5, LPGAT1, LSM3, LSM4, LSM5, LSM8, MDM2 (includes EG:4193), MRPL18, MRPL46, MRPL53, PRKAB1, PWP1, SART3, SPTBN5, SRPRB, STAT1, TMEM49, UFM1, USF1 | 20 | 15 | Gene Expression, Cellular Growth and Proliferation, Hematological System Development and Function |
| 7 | MM8-MM24 | ARFGAP3, C1QTNF6, CBX3, CKB, CLIC4, DDX54, DLEU1, EFNA3, EPHA6, ESR1, GNAI2, HIST4H4 (includes EG:121504), HMGN1, JMJD2A, KDELR3, LAMP1, MYC, NSD1, PCBP2, PIM1, PRDM1, PRDM2, PRSS23, PTPN9, REN, RGS3, RGS18, SHMT1, SLC12A4, TGFA, THY1, TMEM222, TRIM24, Vegf, XBP1 | 19 | 15 | Organ Development, Cancer, Reproductive System Disease |
| 1 | PM8-MM8 | CBX3, CCND1, CDX1, CGN, Creb, CSF3R, CUL4B, DOT1L, DUB, DYRK1A, EZH2, FOXO4, GLI1, GOSR1, GST, Histone h3, ITGA8 (includes EG:8516), L3MBTL, NCAPD2, PITX2, PLOD2, SEC22A, SEC31A, SFRP4, SOD2, SYT9, TFAP2A, TNC, USP3, USP7, USP20, WDR61, WNT2B, ZBTB17, ZEB2 | 41 | 31 | Cancer, Gastrointestinal Disease, Tumor Morphology |
| 2 | PM8-MM8 | AGT, APOA5, C9ORF86, COL1A2, CREM, CTSS, DEAF1, ELAVL1, FAM124B, G alphai, HDAC2, IFI35, IL13RA1, IL2RG, IL4R, IL7R, ING1, LDB1, MDFI (includes EG:4188), P4HA1, PTPN1, RFX2, RGS18, RNF12, RPL30, SEMA4D, SMARCD2, SMARCE1, SPI1, STAT, STAT1, STAT5a/b, Tgf beta, TIAL1, USF1 | 41 | 31 | Cellular Growth and Proliferation, Hematological System Development and Function, Immune Response |
| 3 | PM8-MM8 | Alpha Actinin, BCL10, BFAR, CASP9, CASP10, CASP8AP2, Caspase, CLU, COP I, COPB1, COPB2, COPE, DIABLO, DNAJA1, EEF1A2, FAS, IgG, MARCKSL1, NCF4, NSMAF, PARVG, PSMD2, RCN2, RYK, SFXN3, SNX4, SQRDL (includes EG:58472), TES, TJP3, Tnf receptor, TRADD, TRAF3, TRAF5, XPNPEP3, ZYX | 38 | 30 | Hepatic System Disease, Liver Failure, Organismal Injury and Abnormalities |
| 4 | PM8-MM8 | ALG5, BID, BMP4, BMP15, BMPR1A, C5ORF22, CCND3, CDC42EP3, CDCA4, CDT1, COX1, COX6A1, COX7A2, Cyclin A, Cyclin D, Cytochrome c oxidase, E2f, E2F1, EAPP, EP400, MCM5, OAZ2, PCNA, RAD51, RBL1, RFC4, RHOBTB2, RPA, RRM2, SF3B4, SMARCA5, SRPR, SUV420H1, TTK, UMPS | 38 | 30 | Cell Cycle, Cellular Development, Gene Expression |
| 5 | PM8-MM8 | AFF4, ANAPC2, ANAPC5, ANAPC7, ATF2, ATIC, CD2AP, CUL2, Dgk, DGKG, DGKZ, E3 RING, FGL2, GPN3, IFN Beta, IFNAR2, Interferon beta, IRF, IRF1, IRF7, JARID2, LMNB1, MED13, MED14, MED30, MEP1A, MEP1B, MYBBP1A, PDLIM5, PSTPIP1, Rb, SLC25A13, SPHK1, TARDBP, TMPO | 36 | 29 | Cell Signaling, Gene Expression, Renal and Urological Disease |
| 6 | PM8-MM8 | ANXA1, CALD1, Calmodulin, CCT2, CHEK2, CHGA, Ck2, Cpla2, CTNND1, Cyclin E, DENND4A, FRMD6, FXN, GFAP, GJB1, MAP4K4, P38 MAPK, PLA2, PLA2G6, PLA2G12B, PLA2G4A, PMPCB, PPM1D, PTPN7, RNF11, S100A11 (includes EG:6282), S100B, SLC9A1, TNIP1, TRPM2, TRPV4, WDR77, XPOT, YES1, YWHAB | 36 | 29 | Lipid Metabolism, Small Molecule Biochemistry, Molecular Transport |
| 7 | PM8-MM8 | APBA3, APOB, APP, APPBP2, ARFGEF1, ATP6, ATPase, CHRNG, EIF6, ENTPD2, GRPEL1, HSPA4L, IAPP, Immunoproteasome Pa28/20s, MYO9B, MYOD1, NBL1, PDIA4, PRKAR1A, Proteasome, Proteasome PA700/20s, PSMA, PSMA2, PSMA5, PSMB1, PSMB3, PSMC3, PSMC5, PSMD5, SEC23A, UBE2, UBE2E1, UBE2F, UBE2R2 (includes EG:54926), UBE3A | 35 | 29 | Cellular Assembly and Organization, Neurological Disease, Gene Expression |
| 8 | PM8-MM8 | AIP, Akt, AKTIP, ARNT, ATF4, BAG4, BTRC, CCL20, CRTC1 (includes EG:23373), EHF, ELF3, Esr1-Esr1-estrogen-estrogen, GABBR2, GARS, GCLM, Hdac, HDAC8, HEY2, HLF (includes EG:3131), Hsp70, Hsp90, Mre11, MST1R, NCOR1, NFE2L1, NQO1, NRP1, PAXIP1, PDCD4, PTCD3, RBM9, TARS, TERF2IP, Vegf, XRCC5 | 34 | 28 | Gene Expression, Cellular Growth and Proliferation, Nervous System Development and Function |
| 9 | PM8-MM8 | ANXA5, ARHGAP18, ASPN, Cbp/p300, EIF3B, EIF4E, EIF4G1, FEZ1, FMOD, FUBP1, GPBP1, GTF2A1, GTF2B, HTATSF1, IKBKAP, LPCAT1, MPHOSPH6, MRPS9, N-cor, NELF, NFkB, NR0B2, PARN, RNA polymerase II, RNMT, Rxr, SAR1A, SLC20A1, Smad2/3, TASP1, TDG, TGFB2, Thyroid hormone receptor, TTLL12, VDR | 34 | 28 | Gene Expression, Cellular Assembly and Organization, Skeletal and Muscular System Development and Function |
| 10 | PM8-MM8 | ASF1A, CD3, CD3E, CHAF1B, DOCK1, DOK4, Dynamin, FYN, GRB2, IL6ST, JAK, LYN, OCRL, PACSIN1, PACSIN3, PAFAH1B2, Pdgfr, PI3K, PIK3CA, PIK3R1, PLC gamma, PTPRC, RHOU, SH2B3, SH3GL1, SH3KBP1, SLA, Sos, SOS2, ST5, TCR, TEK, TLK1, VAV, VAV3 | 30 | 26 | Cell Signaling, Cellular Development, Hematological System Development and Function |
| 11 | PM8-MM8 | Actin, ADRB2, CACNA1C, CACNB4, CASP3, CAST, ERC1, ERC2, GZMA, HMGB2, Hsp27, HSPB2, KCNK2, LSP1, MAGI, MAGI3, Mapk, OGG1, p70 S6k, Pkc(s), PP1/PP2A, PP2A, PPP1R7, PPP2R3A (includes EG:5523), PPP2R3B, PPP2R5C, PRKCB1, PTEN, RIMS1, RPS6KB1, SLC6A4, TEAD1, Ubiquitin, VGLL4, XPO6 | 30 | 26 | Cell Morphology, Hematological System Development and Function, Immune and Lymphatic System Development and Function |
| 12 | PM8-MM8 | AFF1, ASAH1, ASAH3, ATP1B3, C6ORF211, CCDC47, CD47, Ceramidase, COX4NB, DPM1, DST, EFR3A, GMPPB, GPX1, GSTK1, HEATR3, HNF4A, Integrin, LGALS3, NUP93, PDCD6IP, RGS1, SECISBP2, SELK, SMAD9, TMCO6, TMEM57, TSC22D1, TSPAN8, TTC35, TXNRD3, UBQLN1, VEPH1, VPS37C, ZNF592 | 23 | 22 | Drug Metabolism, Small Molecule Biochemistry, Lipid Metabolism |
| 13 | PM8-MM8 | CDC123, CHCHD3, COQ3, CTDSPL2, DSCR3, FOXRED1, HNF1A, HNF4A, HPS6, HPS5 (includes EG:11234), MRPS27, PGGT1B, PTK7, RSU1, SORCS3, SUCLG1, TUFT1, TXNL1, ZCCHC8 | 20 | 16 | Genetic Disorder, Gene Expression, Lipid Metabolism |
| 14 | PM8-MM8 | BUD31 (includes EG:8896), C1ORF163, DDX10, FBXO31, GDAP2, GOLIM4, HNF4A, LTA4H, MRPL46, NUDT6, ORMDL2, PCID2, SHFM1, SLC35A1, THYN1, TMEM30A, TMEM63A, UTP23 | 19 | 15 | Cellular Development, Lipid Metabolism, Molecular Transport |
| 1 | MM8-MA8 | ATG5, ATP5J2, BAZ1A, BAZ1B, C3, CBX3, CHAF1A, FTSJ1, GTF2I, HIST1H1E, HNF4A, IRAK1, IRAK4, KIAA0746, L3MBTL, LPGAT1, LPP, MAP3K7, MYO1A, NSD1, PELI1, PRRX1, RARA, RARG, RQCD1, RXRA, RXRB, RXRG, SMARCA5, SMARCE1, SPTBN5, STOML2, TCF12, TLN1, TRAF6 | 22 | 10 | Dermatological Diseases and Conditions, Inflammatory Disease, Cancer |
