# Supplementary material for: Pathway results from the chicken data set using GOTM, Pathway Studio and Ingenuity softwares
Source: BMC Proc. 2009 Jul 16;3(Suppl 4):S11. doi: 10.1186/1753-6561-3-S4-S11 (PMC2712741; doi:10.1186/1753-6561-3-S4-S11)
Supplement: Additional file 2 — Data were filtered with 2 criterions: IPA threshold p.value (<0.05) and the corresponding microarray p.value. a – Biofunctions: we did not find significant functions for the MM8-MA8 gene list. b – Canonical Pathway. For each list, the pathways are ranked by the score (score = -log(p.value)) using the same criterions. The table includes also the ratio (number of focus molecules in a given pathway divided by the total number of the molecules that makes up that pathway). [file 1753-6561-3-S4-S11-S2.doc]

Supplemental data 2 :

Data were filtered with 2 criterions: IPA threshold p.value (<0.05) and the corresponding microarray p.value.

1. Biofunctions: we did not find significant functions for the MM8-MA8 gene list.
2. Canonical Pathway. For each list, the pathways are ranked by the score (score = -log(p.value)) using the same criterions. The table includes also the ratio (number of focus molecules in a given pathway divided by the total number of the molecules that makes up that pathway).

Supplemental data 2 a: IPA bioFunctions

| PM8-MM8 | MM8-MM24 | P-value |
| --- | --- | --- |
| Immune Response |  | 1,08-04/-2,22-02 |
| Immune and Lymphatic System Development and Function |  | 1,08-04/-2,08-02 |
| Endocrine System Development and Function |  | 5,33-04/-1,04-02 |
| Nucleic Acid Metabolism |  | 5,99-04/-1,74-02 |
|  | Endocrine System Development and Function | 6,71-04/-4,1-02 |
| Free Radical Scavenging |  | 1,67-03/-1,67-03 |
| Inflammatory Disease |  | 3,7-03/-3,7-03 |
|  | RNA Post-Transcriptional Modification | 5,54-03/-2,07-02 |
|  | Nucleic Acid Metabolism | 1,44-02/-4,1-02 |
| Viral Function |  | 1,54-02/-1,54-02 |
| Energy Production |  | 2,24-02/-2,24-02 |
|  | Viral Function | 3,76-02/-3,76-02 |

Supplemental data 2 b: IPA canonical pathways

| PM8-MM8 | MM8-MM24 | MM8-MA8 | score | Ratio |
| --- | --- | --- | --- | --- |
|  | Regulation of Actin-based Motility by Rho |  | 2,72 | 7,61-02 |
| Cell Cycle: G1/S Checkpoint Regulation |  |  | 2,12 | 1,38-01 |
| IL-4 Signaling |  |  | 2,09 | 1,29-01 |
| p38 MAPK Signaling |  |  | 2,02 | 1,16-01 |
| Hypoxia Signaling in the Cardiovascular System |  |  | 1,9 | 7,04-02 |
| Interferon Signaling |  |  | 1,85 | 1,72-01 |
|  | Hypoxia Signaling in the Cardiovascular System |  | 1,62 | 1,13-01 |
|  | Synaptic Long Term Potentiation |  | 1,54 | 5,36-02 |
|  |  | Glycosphingolipid Biosynthesis - Lactoseries | 1,5 | 3,57-02 |
| Aminosugars Metabolism |  |  | 1,52 | 7,69-02 |
| Aryl Hydrocarbon Receptor Signaling |  |  | 1,48 | 8,55-02 |
|  |  | N-Glycan Degradation | 1,38 | 3,33-02 |
|  | p38 MAPK Signaling |  | 1,36 | 5,26-02 |
| N-Glycan Degradation |  |  | 1,35 | 1,33-01 |
